# Effectiveness and underlying mechanisms of a group-based cognitive behavioural therapy-based indicative prevention program for children with elevated anxiety levels

**DOI:** 10.1186/1471-244X-13-183

**Published:** 2013-07-05

**Authors:** Manon LA van Starrenburg, Rowella CWM Kuijpers, Giel JM Hutschemaekers, Rutger CME Engels

**Affiliations:** 1 Behavioural Science Institute, Radboud University Nijmegen, Montessorilaan 3, 6525 HR, Nijmegen, Netherlands; 2 Ambulatorium KJJ, Toernooiveld 5, Postbus 6909, 6503 GK, Nijmegen, Netherlands

**Keywords:** Anxiety, Effective elements, CBT, Children, Mediator, Coping, Cognitions, Prevention, Group intervention

## Abstract

**Background:**

Anxiety is a problem for many children, particularly because of its negative consequences not only on the wellbeing of the child, but also on society. Adequate prevention and treatment might be the key in tackling this problem. Cognitive behavioural therapy (CBT) has been found effective for treating anxiety disorders. “Coping Cat” is one of the few evidence-based CBT programs designed to treat anxiety symptoms in children. The main aim of this project is to conduct a Randomized Controlled Trial (RCT) to evaluate the effectiveness of a Dutch version of Coping Cat as an indicative group-based prevention program. The second aim is to gain insight into the mechanisms underlying its effectiveness.

**Methods/design:**

Coping Cat will be tested in Dutch primary school children grades five through eight (ages 7 to 13) with elevated levels of anxiety. This RCT has two conditions: 130 children will be randomly assigned to the experimental (N=65, Coping Cat) and control groups (N=65, no program). All children and their mothers will be asked to complete baseline, post intervention, and 3-month follow-up assessments. In addition, children in both the experimental and control group will be asked to complete 12 weekly questionnaires matched to the treatment sessions. Main outcome measure will be the child’s anxiety symptoms level (SCAS). Four potential mediators will be examined, namely active coping, positive cognitive restructuring, self efficacy and cognitions about ones coping ability (from now on coping cognitions).

**Discussion:**

It is hypothesized that children in the experimental condition will experience reduced levels of anxiety in comparison with the control group. Further, active coping, positive cognitive restructuring, and coping cognitions are expected to mediate program effectiveness. If Coping Cat proves effective as a prevention program and working mechanisms can be found, this group-based approach might lead to the development of a cost-effective program suitable for prevention purposes that would be easily implemented on a large scale.

**Trial Registration:**

Nederlands Trial Register NTR3818.

## Background

Anxiety disorders are known as one of the most common psychiatric disorders in children. Prevalence rates of anxiety disorders in children vary widely from 5% to 23% due to differences in measurements, target groups and recognition of the problems [1-3]. Kessler and colleagues [4] showed that about 75% of anxiety disorders start between 11 years and 21 years of age. Anxiety disorders are also known for their wide range of detrimental consequences. Social competencies, self-esteem, and academic achievement are negatively associated with anxiety disorders [3]. Anxiety in childhood has been associated with greater risk for; more severe anxiety and substance abuse in adulthood [5]. Further, about 30% of the clinically anxious youth have comorbid depression disorder [6]. Altogether, anxiety symptoms in children should be perceived as a serious problem.

The vast majority of children and adolescents with high levels of anxiety do not seek treatment [7]. Partly because of the difficult distinction between normal anxiety in children and symptoms of an anxiety disorder, plus the different manifestation of anxiety in children (more externalizing than internalizing). Underreporting, as well as underdiagnosis, results in a substantial number of children and adolescents with unnoticed and untreated subclinical and clinical anxiety. Indicative prevention might be the key for detecting and treating these symptoms early and preventing future problems.

Meta-analytic review [3] showed that anxiety prevention works best when it’s on indication (only children with elevated anxiety levels participate) focused primarily on anxiety problems and given by well-trained therapists. Furthermore, children under the age of 11 seem to benefit more from prevention programs compared to older children. This pleads for testing a theory-based anxiety program in young children with elevated levels of anxiety. Cognitive behavioural therapy (CBT) is the most effective treatment for anxiety disorders [8,9]. “Coping Cat” is one of the few effective CBT programs for anxiety symptoms in children. This program includes both cognitive techniques (cognitive restructuring and handling physiological responses to anxiety) as behavioural components (relaxation training, exposure). The focus is on exposure, which begins early in the program. Coping Cat has been proved effective in the US [5,10] as well in several other countries, including the Netherlands [11,12]. Although most studies focused on individual treatment, a few studies evaluated a group intervention, which has been found to be just as effective as the individual intervention [13,14]. Because of its effectiveness in clinical samples, therapeutic basis and substantial exposure components, we will test the effectiveness of an indicated group-prevention version of Coping Cat.

Although Coping Cat is effective in decreasing the levels of anxiety and fears, it is still largely unknown what components of this program are essential working elements. To obtain insights into the program’s working mechanisms and eventually optimize the treatment and prevention of anxiety, it is essential to study mediators of change [15]. Beck’s cognitive model assumes that anxious individuals process stimuli in a biased way, resulting in several cognitive errors [16]. Exaggerated perception of danger and an underestimation of one’s ability to cope with these threats are associated with these biased interpretations [17]. An important mediator between experiencing a stressful life event and emotional well-being is coping. Coping is a broad term that includes cognitive, behavioural, and physiological processes and indicates how one responds to stressful situations [18]. Based on several studies, coping skills and self-efficacy are linked to reduction of anxiety symptoms [19]. Maric et al. [20] indicated that one of the strongest predictors of anxiety is the underestimation of the ability to cope. This suggests that cognitions about one’s coping abilities play a mediating role in anxiety treatment. Kendall found some evidence for this claim [21]. Coping is a comprehensive concept, which can be measured using more general as well as more specific strategies. A meta-analysis [22] showed that coping plays a mediating role in treatment gains, regardless of whether it is measured as a general (e.g., self-efficacy, coping strategies) or a specific strategy (coping cognitions). We aim to test whether active coping, positive cognitive restructuring, self-efficacy, and cognitions about coping ability mediate the effect of CBT treatment on anxiety in children.

### Aim and hypothesis

The main aim of this project is to conduct a Randomized Controlled Trial (RCT) to evaluate the effectiveness of a CBT based program called Coping Cat on the anxiety levels of Dutch primary school children. Therefore, Coping Cat will be revised and used as an indicative prevention group-based program. In this RCT, we will assign children to an experimental and a control group to test whether children who received Coping Cat as an indicated-group prevention program will have lower levels of anxiety at follow-up measurements. The second aim is to determine whether active coping, positive cognitive restructuring, self efficacy and coping cognitions operate as mediators. More specifically, we expect that a) anxiety levels of children in the prevention program will decrease significantly more over time compared to anxiety levels of children in the control group. b) Children who will follow the program will have significantly enhanced coping skills compared to the control group.

## Methods/design

### Study design

**Figure 1:**
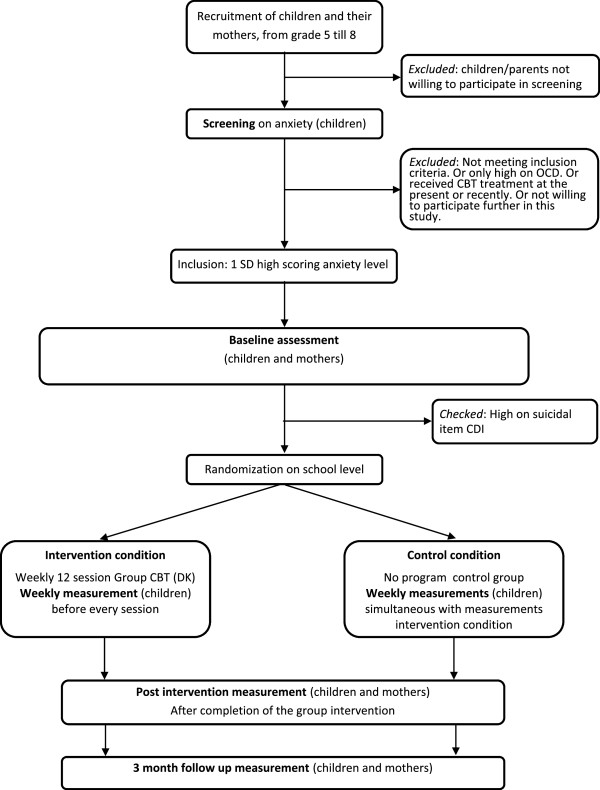
Study design.

Coping Cat will be adjusted and tested as an indicated CBT-based prevention program in Dutch primary school children, grades five through eight, with elevated levels of anxiety. This study is set up as a RCT with two conditions, with 130 children being randomly divided into the experimental (N=65) and control group (N=65). The experimental group will receive the adapted 12-session Coping Cat program. The control group will not receive a program. They will be offered the possibility to follow the program at the end of the study. Participating children and their mothers will complete baseline, post intervention, and a 3-month follow-up assessments (see Figure 1). In addition, children in both experimental and control group will be asked to complete questionnaires weekly, just before every program session. All mothers who will complete all questionnaires will receive about 50 Euros for their participation. Children will receive few small for their participation.

### Participants

#### Recruitment

Children will be recruited from a sample of primary schools in the Nijmegen-region in the Netherlands. Schools will be asked to participate and distribute a letter to all children from grades five till eight, in which we will inform children and parents about this study. Parents who do not want their child to participate in the screening can return an enclosed objection form. After screening, all parents will be informed about their child’s anxiety level, as revealed by the screening questionnaire. Parents whose children will have significantly increased levels of anxiety will be contacted by telephone. This will done to assure that they understand the implications and limitations of these results. In contrast to the passive consent procedure regarding the screening, active consent will be obtained for children’s participation in the intervention and control group. By signing and returning an attached consent form, parents will provide permission for their child(ren) to participate in one of the groups. This study received approval from the Faculty Ethics Committee.

#### Eligibility criteria

To be included in the screening, children will have to be in grades 5–8 in one of the participating Dutch elementary schools. Moreover, the children as well as their parents will have to speak and read Dutch to be able to complete the questionnaires and participate in therapy sessions. To participate in the RCT, children will have to report their levels of anxiety. This will be measured using an anxiety questionnaire (Spence Children’s Anxiety Scale) [23]. All children who will score above a 1 SD cut off score will be included. In addition, children who will score above the cut off score on the obsessive-compulsive disorder scale only will not be able to participate in the study. Children with OCD are known to respond better to specific CBT treatment [24]. Parents of children who will score high on suicidal ideation (1 item, Child Depression Inventory) [25] will be contacted by telephone by an experienced therapist to make sure this information is communicated carefully. Subsequently, it will be decided whether the child can participate in the study or whether referral to mental health care is needed. Children who followed CBT during the preceding year or will be following a treatment with a CBT basis will not be able to participate in the study. When the child already has knowledge on specific cognitive techniques, this will interfere with determining the underlying mechanisms. Children with diagnosed comorbidity, such as ADHD, ODD, and the like, will be eligible.

#### Randomization

Randomization will be applied within schools, with an equal number of experimental and control groups within one school. Children will be assigned to either the treatment or the control condition. Participants will be stratified by age (young: 7–9 years. & old: 10–12 years) and number (equally distributed over both conditions).

#### Sample size calculation

Based on the outcomes of Coping Cat effect studies abroad and in the Netherlands, a high effect size is expected [11,13]. However, all these children were residing in a child welfare institution and diagnosed with an anxiety disorder. The anxiety problems are thus expected to be more severe in these children compared to the participants in the current study. Therefore, the effect of the intervention in this study is expected to be smaller. Research on indicated anxiety prevention [3] has shown that effect sizes around .32 can be expected. To be on the safe side, we estimated a medium effect of F^2^ .15 at three months follow-up, and further increased the sample to account for clustering of the data, which lead to 65 participants per condition. About 30% of the children are expected to have elevated levels of anxiety, with an expected response rate of 60% of these children with elevated levels of anxiety participating, about 700 children should be approached.

### Program intervention

A Dutch Coping Cat group therapy program will be used as an indicated prevention. We adjusted a Dutch version of the individual orientated program [26] according to the content of the American group therapy version of Coping Cat [27]. The main changes concern the increased duration of the program (from 18 to 12 sessions), increased number of group elements, and decreased contact time (from 2 hours to 1 hour). Moreover, the exposure exercises will start earlier in the program (session 4 instead of session 8).

#### Intervention condition

Children randomly selected to the intervention condition will receive a 12-session group program, each session lasting 1 hour. The sessions will take place every week, except for holidays. Groups consist of 7 to 9 children. The program will take place in schools after regular school hours. The program will start by using exercises enhancing group cohesion and safety. These exercises will continue throughout the program. In session 2 and 3, psycho-education about anxiety will be given and anxious feelings, both physical and emotional, will be addressed. Moreover, a relaxation exercise will be introduced. Children will be motivated to keep rehearsing relaxation every day during several weeks. The next step (session 4) will be to recognize thoughts and stimulate positive thinking. Meanwhile, exposure will be introduced beginning with session 4. Children will practice different anxiety provoking situations as homework at home, thus in low anxiety situation. In group therapy, children learn to make a plan when entering anxious situations (session 5). Beginning with session 6, children will experience anxious situation during therapy. They will start easy, being exposed to low-level anxiety situations as a group and slowly experiencing high-level anxiety situations that they will face alone. Meanwhile, children will repeat the techniques they acquired. Session 12 will end with an evaluation and little party to celebrate the children’s results. Parents will receive written information three times during the program, with specifics about the progress of their child and general information about the program.

#### Trainers

Trainers (n=5) are child psychologists with ample experience in youth mental health care and knowledge of CBT. All trainers will participate in two-day training. In this training, the protocol will be explained and trainers will complete exercises and role-plays. Therapists will follow 2-h supervision sessions three times over the course of the program. A MA student in clinical child psychology will assist each therapist.

#### Control condition

Children in the control group will be asked to complete questionnaires weekly while children in the program will be completing these questionnaires. Children will be administered the Coping Cat directly after the 3-month follow-up assessment.

### Data collection

Children will receive all questionnaires on paper and complete them at school, after school hours, in the presence of one of the researchers or trainers. This context is chosen deliberately to exclude any influence of parents. Mothers will complete the pre-, post-, and follow up measurement online. For this purpose, they will receive separate personal login codes that will give them access to their own questionnaire on a secured webpage. Mothers who prefer a paper questionnaire will be provided with a paper and pen version. Interference of the researcher will be diminished as much as possible by presenting the questionnaires digitally. Children and mothers are expected to complete the pre-, post-, and follow up assessments within 45 minutes. The weekly measurements will take about 10 minutes to fill out. Non-responding mothers and absent children will be contacted by phone and motivated to complete the questionnaire on paper or directly by phone. An overview of all measures at each time point is provided in Table 1.

**Table 1:** Overview of measures

**Measure**	**Screening**	**Baseline**		**Weakly**		**Post treatment**		**3 month follow up**	
	**Child**	**Child**	**Mother**	**Child**	**Therapist**	**Child**	**Mother**	**Child**	**Mother**
Spence Children's Anxiety Scale (SCAS)	X	X	X			X	X	X	X
Multidimensional Anxiety Scale for Children (MASC- 10)		X		X		X		X	
Suicidal item Child Depression Inventory (CDI)		X				X		X	
Coping Strategies For Children^1^ (CSLK)		X		X		X		X	
The Self-Efficacy Questionnaire for Children^2^ (SEQ-C)		X		X		X		X	
Coping Questionnaire Child (CQ-C)		X		X		X		X	
Therapeutic Alliance Scale for Children (TASC)				Session 1/6/12	X				
Demographic characteristics		X	X						
5 most anxious situations (for treatment purposes)		X							

^1^ Subscales used: Active coping & Positive cognitive restructuring.

^2^ Subscale used: Emotional self efficacy.

#### Outcome

Primary outcome measure is the children’s anxiety level, which will be measured with the Dutch version of the Spence Children’s Anxiety Scale (SCAS). The SCAS was developed to assess anxiety symptoms in children in the general population. The SCAS consists of six subscales, namely panic attack and agoraphobia, separation anxiety disorder, social phobia, physical injury fears, obsessive compulsive disorder and generalized anxiety disorder. The child version consist of 44 items on a *never* (0) to *always* (3) scale. The parent-report (P) version consist of 38 items in the scale on the same *never* (0) to *always* (3) scale. Six positive filler items of the children’s SCAS, were not included in the parent version. The items of the SCAS-P were formulated as closely as possible to the corresponding item of the child version of the SCAS. The SCAS shows high internal consistency, not only for the total scale, but also for each subscale [23,28].

Secondary outcome measures will be active coping and positive cognitive restructuring, measured with the Dutch version of the Coping Strategies Checklist for Children (CSLK). This questionnaire is a children’s self-report measurement of coping skills, and consist of five subscales of which two subscales (active coping and positive cognitive restructuring) are included. The CSLK as used in this study consists of 24 statements which all start with “If I have a problem” followed, for example, by a statement such as “I tell others how I would like to solve it”. The children could choose between four reactions form *never* (1) to *always* (4). The CSLK shows high internal consistency [29]. Cognitions about ones coping skills will be measured with the Dutch version of the Coping Questionnaire- Child (CQ-C) [21], wherein children rated their ability to cope on a 7 point scale that ranged from *not at all able to help myself feel comfortable* (1) to *completely able to help myself feel comfortable* (7). The Self-efficacy Questionnaire for Children (SEQ-C) represents three domains of self-efficacy: social self-efficacy, academic self-efficacy and emotional self-efficacy. Only the latter is included in this study. The emotional self-efficacy scale consists of 8 items and pertains to the perceived capability of coping with negative emotions. Each item has to be scored on a 5-point scale with *not at all* (1) to *very well* (5) [30].

Other outcomes are therapeutic alliance, measured with the Dutch translated version of the Therapeutic Alliance Scale for Children (TASC-nl). Wherein positive and negative aspects of the therapeutic alliance are measured. The TASC-nl includes 12 items on a 4-point scale, ranging from *not at all* (1) to *very much* (4). In previous research the TASC has demonstrated adequate internal consistency [31]. Also anxiety symptoms in children are measured weekly by the Dutch version of the short Multidimensional Anxiety Scale for Children (MASC-10) [32]. A 10-item self-report questionnaire, wherein items are rated on a 4-point scale ranging from *never true about me* (1) to *often true about me* (4). An overview of all measures at each time point is provided in Table 1.

#### Statistical analysis

In accordance with the intent-to-treat principles, all children randomized to a condition will be included in the analyses to test the study hypotheses. Moreover, while randomization takes place within school level and children are ‘nested’ within these schools, we need to control for clustered data [33]. Mplus is a statistical software program that has special features to deal with missing data and it allows analyzing complex data while considering their clustered structure. The 3-month follow up measurement of children’s anxiety levels will be used as the main outcome. Regression analyses will be conducted to test whether children in the experimental condition show a stronger decrease in anxiety symptoms at 3-months follow up compared to the control condition. The effect sizes as well as confidence intervals will be reported to determine both the magnitude and the effect of Coping Cat in the form of an indicative group-based prevention program. We are also interested in possible mediators in the relation between Coping Cat prevention program and anxiety levels. Using LGM in Mplus, we will test whether active coping, positive cognitive restructuring, and cognitions about ones coping ability function as mediators of treatment outcomes.

## Discussion

This study is designed as a RCT to evaluate the effectiveness of Coping Cat as a group-based cognitive behavioural therapy-based indicative prevention program for 7 to 13 years old Dutch children with elevated anxiety levels. It is hypothesized that children in the experimental condition will experience significantly reduced levels of anxiety compared to the control group. Further, active coping, coping cognitions, and positive cognitive restructuring are expected to mediate the program effectiveness.

### Strengths and limitations

Strength of this study is the real-world conditions under which this study is implemented, the program will be delivered in a school setting, with entire schools participating. Which is important for its practical implementation. An additional strength of the study is that most RCT studies focus solely on the effectiveness of the tested program but do not examine how the intervention works (i.e., mediators of change). This study’s second aim is to gain insight into the mechanisms underlying its effectiveness, by testing whether coping mediates the treatment outcome. Rather unique in our design is a session-by-session measurement of the mediators. As a result, we hope to be able to closely monitor differences in coping and possibly provide direction towards pinpointing effective elements within CBT. However, more research on this topic would be necessary to be able to clearly identify working elements in CBT treatment. Second limitation is that by using a within-school design (as opposed to a between-schools design), contamination effects between the experimental and the control group could occur.

### Implications for practice

If we find that Coping Cat can be effective as an indicated prevention program this group-based approach might result in a cost-effective program that is suitable for prevention purposes and can be easily implemented on a large scale. In addition, it can be used not only for prevention purposes, but also for treatment in clinical settings. In the Netherlands, there is a need for an evidence- based (group) program for the treatment of anxious children. Due to the group approach, it might be more cost effective compared to an individual treatment.

## Conclusion

This study will evaluate the effectiveness of a group-based cognitive behavioural therapy-based indicative prevention program for children with elevated anxiety levels. The results of this study will provide insights into the effectiveness of the Coping Cat program and test its underlying mechanisms.

## Competing interests

The authors declare that they have no competing interests.

## Authors’ contributions

MvS is responsible for the data collection, data analysis and for reporting the study results. All other authors are supervisors and grant applicators. All authors read and approved the final manuscript.

## Pre-publication history

The pre-publication history for this paper can be accessed here:

http://www.biomedcentral.com/1471-244X/13/183/prepub
